# Effect of β-sitosterol on insulin resistance & protein expression of insulin signalling molecules in quadriceps muscle of high fat diet-induced type-2 diabetic rats

**DOI:** 10.6026/973206300181098

**Published:** 2022-11-30

**Authors:** Gayathri Sekar, Merlin G, Madhan Krishnan, Shyamaladevi Babu, Padmini K, Pushpa Konduru, Mangai S

**Affiliations:** 1 PG and Research Department, Department of Biochemistry, Bhaktavatsalam Memorial College for Women, Korattur chennai-600080, Tamilnadu, India; 2 Department of pathology, Biosciences research Foundation, Chennai, Tamilnadu, India; 3 Department of Biostatistics, ACS Medical College and Hospital, Velappanchavadi, Chennai 600077, Tamilnadu, India; 4 Faculty of Allied Health Sciences, Chettinad Hospital and Research Institute, Chettinad Academy of Research and Education, Kelambakkam-603103, Tamilnadu, India; 5 RCC Laboratories India Private Limited, Chennai, Tamilnadu, India

**Keywords:** β–sitosterol, high fat diet, insulin resistance, IR, GLUT4, type-2 diabetes

## Abstract

Changes in life style, such as high-calorie diet intake and lack of exercise, have increased the global prevalence of obesity and diabetes. The major pathophysiological event contributing to the development of type 2 diabetes mellitus is the target tissues to insulin action. Currently available drugs are unsuccessful for the treatment of type 2 diabetes due to their unwanted side effects. Hence, search drugs, from plant sources. β-sitosterol is plant sterols with structurally almost like that of cholesterol. It is widely present in various medicinal plants. It has been reported to elicit multitude of bioactivities including anti-lipidemic and anti-hyperglycemic activity. However, specific effect of β-sitosterol on insulin signalling molecules of quadriceps muscle remains unclear. Hence, the study aimed to assess the beneficial role of β-sitosterol on the expression of insulin signalling molecules in quadriceps muscle of high fat diet and sucrose-induced type-2 diabetic rats. The oral effective dose of β-sitosterol (25 mg/kg body weight) was given once each day until the end of the study (30 days post‑induction of diabetes) to HFD- fed diabetic rats. At the end of the experimental period, fasting blood sugar (FBG), oral glucose (OGTT) and insulin tolerances (IT), Tissue glycogen, Serum insulin, Homeostasis Model Assessment for Insulin Resistance (HOMA-IR) and Quantitative Insulin Sensitivity Check Index (QUICKI), as well as the levels of insulin signalling molecules like insulin receptor (IR), glucose transporter subtype 4 (GLUT4) proteins and glycogen concentration within the quadriceps muscle were assessed. A diabetic rat indicates impaired glucose and insulin tolerances and insulin signalling molecules (IR and GLUT4) proteins and glycogen concentration. Serum insulin, were found to be increased in diabetic rats. The treatment with β-sitosterol normalized the altered levels of blood glucose, serum insulin, and IR and GLUT4 protein levels. Thus, we concluded that β-sitosterol enhances glycemic control through activation of IR and GLUT4 in the quadriceps muscle of high fat diet and sucrose‑induced type2 diabetic rats.

## Background:

Diabetes mellitus is defined by aberrant serum insulin levels or insensitivity of target tissues to insulin action and is associated with improper carbohydrate, lipid, and protein metabolism [1].Diabetes affects 425 million people worldwide in 2017, with the number expected to rise to roughly 629 million by 2045 [2].Diabetic ketoacidosis, non-ketotic hyperosmolar coma, and diabetic coma are the acute consequences of diabetes. The chronic complication is caused by prolonged blood sugar rise, which damages blood vessels and causes dysfunction and failure of numerous organs, particularly the eyes, kidneys, nerves, and heart [3]. The currently available medications for diabetes control have some drawbacks, necessitating the search for safer and simpler anti-diabetic drugs [4].The development process in anti-diabetic medication discovery has moved its focus to plant-derived therapies due to their safety, efficacy, cultural acceptability, and lower side effects [5].β-sitosterol is a common plant sterol found in many different plants. It has been used as a food ingredient in processed foods due to its nutraceutical properties. Many investigations have been conducted to investigate the pharmacological activities of β-sitosterol [6]. There have been no researches on β-sitosterol anti-diabetic efficacy on quadriceps muscles. However, the mechanisms behind β-sitosterol anti-diabetic activity on insulin signal transduction in quadriceps muscle are largely unclear. As a result, the current study aims to elucidate the function of β-sitosterol on insulin signalling molecules inside the quadriceps muscle of type 2 diabetic rats fed a high fat diet.

## Materials and Methods:

## Chemicals:

All chemicals and reagents used in this investigation were obtained from Sigma Chemical Company (St. Louis, MO, USA); Invitrogen (USA); Eurofins Genomics India Pvt Ltd (Bangalore, India); New England Biolabs (NEB) (USA); Promega (USA); Santa Cruz Biotechnology (USA) and Cell Signaling Technology (USA).β-actin monoclonal antibody was bought from Sigma (USA). Total RNA isolation reagent (TRIR) was obtained from Invitrogen, USA. The reverse-transcriptase enzyme was bought from New England Biolabs (NEB) (USA)and Go Taq Green master mix was obtained from Promega (USA). Insulin receptor (IR), insulin receptor substrate-1 (IRS-1), Akt, glucose transporter-4 (GLUT4) and β-actin primers were purchased from Eurofins Genomics India Pvt Ltd (Bangalore, India) and Polyclonal IR and GLUT4 antibodies were purchased from Santa Cruz Biotechnology, Inc. (Santa Cruz, C.A).

## Animals:

Animals were maintained as per the National Guidelines and Protocols approved by the Institutional Animal Ethics committee (IAEC No: 011/2016 dated 04.07.2016). Healthy adult male albino rats of Wistar strain (Rattusnorvegicus) (150-180 days old weighing 180-200g were used in this present study and maintained in clean polypropylene cages at the Central Animal House Facility, Meenakshi Medical College and Research Institute (Meenakshi Academy of Higher Education and Research) under particular humidity (65 ± 5 %) and temperature (21 ± 2°C) with constant 12 h light and 12 h dark schedule. They were fed with standard rat pelleted diet (Lipton India, Mumbai, India), and clean drinking water was made available ad libitum.

## Induction of type-2 diabetes:

Rats were fed with HFD composed of, 1% cholic acid, 3% cholesterol 30% coconut oil, and 66% standard rat feed for 60 days. Also, they were concurrently fed with 30% sucrose through drinking water. On the 58th day of treatment period, after overnight fasting, blood glucose was inspected and the rats with blood glucose level >120mg/dl were selected as type-2 diabetic rats. Rats were fed with HFD and sucrose water until end of the study.

## Experimental design:

The following experimental design was framed and accordingly the rats were subjected to treatment for a period of one month. Healthy adult male Wistar rats were divided into the following groups of 6 rats each.

Control and experimental animals were given an oral glucose tolerance test (OGTT) and an insulin tolerance test (ITT) two days before they were sacrificed. After 30 days, blood was drawn and the animals were perfused with physiological saline while anaesthetized with sodium thiopentone (40 mg/kg b.wt) and quadriceps muscle was dissected out to evaluate various properties.

##  Fasting blood glucose (FBG):

Blood glucose was evaluated using On-Call Plus blood glucose test strips (ACON Laboratories Inc., USA) after overnight fasting. Blood was collected by nicking the tip of the rat tail and results were expressed as mg/dl.

##  Oral Glucose Tolerance Test (OGTT):

For oral glucose tolerance test, rats were fasted overnight and blood glucose was evaluated using On-Call Plus blood glucose test strips at various time periods (60, 120 and 180 min) after providing the oral glucose load (10 ml/kg; 50% w/v). Blood glucose value before giving glucose load was considered as 0 minute value. Results were expressed as mg/dl.

## Insulin Tolerance Test (ITT):

This test was performed on random-fed rats. Animals were injected with insulin (0.75 U/kg) in ∼ 0.1 ml 0.9% saline intra peritoneal. A drop of blood (5µl) was taken from the tail vein before the injection of insulin and after 15, 30, 45, and 60 min for the conformation of blood glucose with a glucometer. Results were expressed as mg/dl.

## Homeostasis Model Assessment for Insulin Resistance (HOMA-IR) and Quantitative Insulin Sensitivity Check Index (QUICKI):

HOMA-IR was calculated utilize the formula (fasting blood glucose X fasting serum insulin/405) as per the method of Matthews et al. 1985 and QUICKI was calculated utilize the formula 1/ (log fasting serum insulin + log fasting blood glucose) as per the method of (Katz et al. 2000) [7].

## Fasting serum insulin:

Serum insulin was assayed using ultrasensitive rat insulin ELISA kit obtained from Crystal Chem Inc (Illinois, USA). The range of detection is 0.1 - 64 ng/ml. The percentage cross-reactivity of insulin antibody to rat insulin was 100%. Intra-assay coefficient of variation was ≤10.0% and inter-assay coefficient of variation was <10.0%. Results were expressed as ng/ml.

## Tissue glycogen:

Glycogen was estimated by the method of Hassid and Abraham 1957 [8]. Results for the amount of glycogen concentration were communicated as mg/g wet tissue.

## Protein expression analysis:

## Protein isolation and western blotting:

100 mg of adipose tissue from control and experimental animals were used to isolate proteins. 1 ml of buffer A (5 mM NaN3, 0.25 M sucrose, 10 mM NaHCO3) was added to 100 mg of adipose tissue, homogenized, and centrifuged at 1300xg at 4°C for 10 minutes. The supernatant was separated and centrifuged at 12,000xg for 15 minutes at 4°C. To evaluate the post-receptor insulin signaling molecules, the final supernatant was sampled as a total protein. The protein estimation was done using the Lowry et al. [9] technique.

The lysate proteins (50g/lane) were isolated and electro blotted onto a poly vinylidene di fluoride (PVDF) membrane (Bio-Rad Laboratories Inc) using sodium dodecyl sulfate-polyacrylamide gel electrophoresis (10 % gel). The membranes were blocked with 5% non-fat dry milk and tagged with primary antibodies (1:1000 dilutions). After three washes with TBS-T, the membrane was incubated for 1 hour with a 1:5000 dilution of horseradishperoxidase-conjugated rabbit-anti-mouse or goat-anti-rabbitsecondary antibody (GeNei, Bangalore, India). Following theincubation period, the membrane was washed three times with TBSand TBS-T. The protein bands were visualized using a sophisticatedChemiluminescence detection system (Thermo Fisher Scientific Inc.,Waltham, MA, USA), the specific signals were found, and protein bands were captured and quantified using Chemidoc and QuantityOne image analysis systems from Bio-Rad Laboratories, CA. Themembrane was then stripped for 30 minutes at 50°C in strippingbuffer (50 ml, 62.5 mMTris–HCl (pH 6.7), 1 g SDS, and 0.34 ml –mercapto ethanol). The membranes were then reprobed using ananti β -actin antibody (1:5000). The invariant control used was β -actin. 

## Statistical analysis::

Using one-way analysis of variance (ANOVA) and Duncan'smultiple range tests, computer-based software, the data wereanalyzed to determine the significance of individual variancewithin the control and treated groups (Graph Pad Prism version 5).Duncan's test was used to determine significance at the level ofp<0.05.

## Results and Discussion:

Diabetes mellitus is a chronic metabolic disorder characterized byinsufficient insulin secretion, or resistance to insulin action, or both.It is associated with abnormalities in carbohydrate, lipid andprotein metabolism, which leads to hyperglycemia, hyperlipidemia, hyperinsulinemia and hypertension [10]. Management of diabetesmellitus normally involves diet, exercise and chemotherapy. The conventional pharmacological treatments are associated with many adverse side effects and high rates of secondary failure which lead to an increasing demand for natural products with anti-diabetic activity and lesser side effects [11]. Thus, it is important to look for more potent anti-diabetic agents preferably from dietary sources, which should be cost effective and have lesser or no side effects. Hence trends on analysing the hypoglycemic effect of phytosterol gained great interest due to its abundance in plants and efficacy.β- sitosterol is a well-known natural sterol that elevates enzymatic and non-enzymatic antioxidants in cells making it an effective anti-diabetic, neuro protective, and chemo protective agent [12]. The high potential of this compound and its analogs in the treatment of various illnesses classifies this compound as the noteworthy drug for the future. Hence, β-sitosterol was used in our study to reveal the mechanism of action of β- sitosterol on the expression of IR and GLUT4 in quadriceps muscle of high fat diet and sucrose induced type-2 diabetic rats.

Insulin resistance in skeletal muscle is a primary and important phenomenon in the development of type-2 diabetes. Up to 85% of whole body insulin-stimulated glucose uptake occurs in skeletal muscle and it is mediated by the translocation of glucose transporter molecules, mainly glucose transporter-4 (GLUT4) from endoplasmic reticulum to the plasma membrane [13]. Many intracellular signaling cascades are involved in the translocation of GLUT4 vesicles including phosphorylation of insulin receptor substrate (IRS) molecules, phosphatidylinositol-3- kinase and protein kinase B (or) Akt [14]. In the present study, high-fat diet and sucrose feeding led to a central adiposity in experimental rats which resulted in destruction of the insulin action and disarrayed the regulation of plasma glucose, in turn leading to hyper glycemia. Epidemiological studies support that the concept of hyper glycemia is the main key factor resulting in the array of diabetic complication [15]. So in the present study reduction of plasma glucose was tested primarily in diabetic rats. Results of our study showed that β-sitosterol revealed the positive effects through decreasing the plasma glucose levels (Figure 1 - see PDF) either of the two mechanisms or collective effect of both i.e. due to the reduction in the intestinal glucose absorption or elevation in the glycolytic and glycogenic pathways with associated decrease in the glycogenolysis and gluconeogenesis pathways. It suggests that this has effects on the glucose metabolic pathways. The acute elevation of plasma insulin (Figure 5 - see PDF) in high-fat diet fed type-2 diabetic rats observed in the present study probably reflects an attempt by β-islet cells to compensate against hyperglycemia. β- sitosterol restored the insulin to the normal level which suggests that insulin-like property as explained in rat skeletal muscle (L6- myotubes) [16].

Oral glucose tolerance test (OGTT) is the most common and highly sensitive for early abnormalities in the regulation of glucose than fasting plasma glucose and HbA1C [17]. The data obtained in OGTT (Figure 2 - see PDF) also confirms the anti-hyperglycemic potential of β- sitosterol. In the present study, the diabetic rats showed elevated levels of glucose even after 2h. Whereas, in β- sitosterol treated diabetic rats; glucose levels were returned to fasting values after 2h. In addition, Insulin resistance (Figure 3 - see PDF) and insulin sensitivity index values clearly depict a severe insulin resistance in diabetic animals. When compared to control, a significant increase in HOMA-IR and decrease in QUICKI values (Table 5 - see PDF) were observed in diabetic animals, whereas administration of β-sitosterol significantly altered these parameters to normal range. A significant decrease in glycogen concentration (Figure 4 - see PDF) in the quadriceps muscle was also observed in high fat and sucrose-fed rats, which may be attributed to impairment in glycogenesis due to insulin resistance. β-sitosterol treated diabetic rats showed a significant increase in glycogen content.

Insulin resistance in skeletal muscle is the primary defect before the β cell dysfunction and hyperglycemia [18]. When insulin binds with its receptor (IR) it activates the receptor tyrosine kinase, which in turn phosphorylates and engages other IRS proteins. Tyrosine phosphorylated IRS provide binding sites for phospatidylinositol 3 kinase (PI3K) which in turn, activates Akt/protein kinase B, resulting in increased translocation of intracellular GLUT4 to the plasma membrane. The activation of the IRS, PI3K, and Akt pathway facilitates glucose uptake by the skeletal muscle cells [19]. Insulin mediated glucose transport is decreased in the skeletal muscle during insulin resistant states such as obesity, hypertension and type 2 diabetes. This is due to impairment in the expression and functionality of the insulin signalling pathway [20]. The present study showed a significant decrease in the IR protein expression in quadriceps muscle (Figure 6 - see PDF) of diabetes induced rats. β-sitosterol treated type 2 diabetic rats showed increased IR protein levels as a result of hypolipidemic potential of β-sitosterol

The uptake of glucose in insulin sensitive tissues, such as skeletal and quadriceps muscle and adipose tissue is mediated by GLUT4 transporter. When insulin binds with its receptor, GLUT4 vesicles are translocated from the cytoplasm to the plasma membrane and mediates glucose uptake by cells. Insulin resistance in type 2 diabetes is due to decreased translocation of GLUT4 [21]. In our study, diabetes-induced rats showed significant decrease in the GLUT4 protein expression in both the cytosolic fractions (Figure 7 - see PDF) and plasma membrane (Figure 8 - see PDF). The increased FFA levels during type-2 diabetes may reduce the expression and translocation of GLUT4 from cytosol to plasma membrane. The expression of GLUT4 promoter in cardiomyocytes and GLUT4 protein in human cardiac muscle biopsies were reduced during increased FFA and lipotoxicity condition, which also attenuated the insulin signaling and GLUT4 translocation through activation of the IkB kinase (IKK) pathway [21]. However β-sitosterol treated diabetic rat's increase the GLUT4 levels in both plasma membrane and cytosol may be due to β-sitosterol mediated increase in insulin signaling molecule (IR). This study clearly shows the antidiabetic potential of β-sitosterol.

## Conclusion:

The current study on the protective effect of β-sitosterol on the protein expression of insulin signalling molecules such as IR and GLUT4 in high fat diet induced type-2 diabetic rats elucidated the anti-diabetic potential of β-sitosterol and could be used as a therapeutic agent in the management of type-2 diabetes, which is a growing major health problem worldwide with a significant financial burden on society.
